# Poly[(μ_6_-4-amino-3,5,6-trichloro­pyridine-2-carboxyl­ato)aqua­caesium]

**DOI:** 10.1107/S1600536812049562

**Published:** 2012-12-08

**Authors:** Graham Smith

**Affiliations:** a Science and Engineering Faculty, Queensland University of Technology, GPO Box 2434, Brisbane, Queensland 4001, Australia

## Abstract

In the structure of the title complex, [Cs(C_6_H_2_Cl_3_N_2_O_2_)(H_2_O)]_*n*_, the caesium salt of the commercial herbicide picloram, the Cs^+^ cation lies on a crystallographic mirror plane, which also contains the coordinating water mol­ecule and all non-H atoms of the 4-amino-3,5,6-trichloro­picolinate anion except the carboxyl­ate O-atom donors. The irregular CsCl_4_O_5_ coordination polyhedron comprises chlorine donors from the *ortho*-related ring substituents of the picloramate ligand in a bidentate chelate mode, with a third chlorine bridging [Cs—Cl range 3.6052 (11)–3.7151 (11) Å] as well as a bidentate chelate carboxyl­ate group giving sheets extending parallel to (010). A three-dimensional coordination polymer structure is generated through the carboxyl­ate group, which also bridges the sheets down [010]. Within the structure, there are intra-unit water O—H⋯O_carboxyl­ate_ and amine N—H⋯N_pyridine_ hydrogen-bonding inter­actions.

## Related literature

For background information on picloram, see: Mullinson (1985 ▶); O’Neil (2001 ▶). For examples of structures of metal complexes with picloram, see: Smith *et al.* (1981*a* ▶,*b* ▶); O’Reilly *et al.* (1983 ▶). For another structure with caesium cations involving coordinating carbon-bound Cl, see: Levitskaia *et al.* (2000 ▶). For a caesium complex with dipicolinic acid, see: Santra *et al.* (2011 ▶).
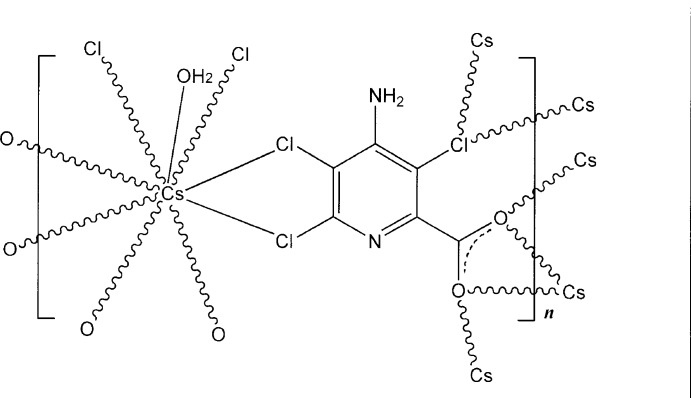


## Experimental

### 

#### Crystal data

[Cs(C_6_H_2_Cl_3_N_2_O_2_)(H_2_O)]
*M* *_r_* = 391.37
Monoclinic, 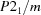

*a* = 7.0816 (3) Å
*b* = 6.6863 (2) Å
*c* = 11.7382 (5) Å
β = 101.005 (4)°
*V* = 545.58 (4) Å^3^
*Z* = 2
Mo *K*α radiation
μ = 4.11 mm^−1^
*T* = 200 K
0.25 × 0.20 × 0.08 mm

#### Data collection

Oxford Diffraction Gemini-S CCD detector diffractometer
Absorption correction: multi-scan (*CrysAlis PRO*; Agilent, 2012 ▶) *T*_min_ = 0.67, *T*_max_ = 0.98
3773 measured reflections
1164 independent reflections
1118 reflections with *I* > 2σ(*I*)
*R*_int_ = 0.026

#### Refinement

*R*[*F* ^2^ > 2σ(*F* ^2^)] = 0.021
*wR*(*F* ^2^) = 0.053
*S* = 0.98
1164 reflections
89 parameters
H-atom parameters constrained
Δρ_max_ = 0.55 e Å^−3^
Δρ_min_ = −0.56 e Å^−3^

### 

Data collection: *CrysAlis PRO* (Agilent, 2012 ▶); cell refinement: *CrysAlis PRO*; data reduction: *CrysAlis PRO*; program(s) used to solve structure: *SIR92* (Altomare *et al.*, 1993 ▶); program(s) used to refine structure: *SHELXL97* (Sheldrick, 2008 ▶) within *WinGX* (Farrugia, 2012 ▶); molecular graphics: *PLATON* (Spek, 2009 ▶); software used to prepare material for publication: *PLATON*.

## Supplementary Material

Crystal structure: contains datablock(s) global, I. DOI: 10.1107/S1600536812049562/wm2705sup1.cif

Structure factors: contains datablock(s) I. DOI: 10.1107/S1600536812049562/wm2705Isup2.hkl

Additional supplementary materials:  crystallographic information; 3D view; checkCIF report

## Figures and Tables

**Table 1:** Selected bond lengths (Å)

Cs1—Cl5	3.7151 (11)
Cs1—Cl6	3.6052 (11)
Cs1—O1*W*	3.129 (3)
Cs1—O21^i^	3.116 (2)
Cs1—O21^ii^	3.116 (2)
Cs1—O21^iii^	3.150 (2)
Cs1—O21^iv^	3.150 (2)
Cs1—Cl3^v^	3.7127 (4)
Cs1—Cl3^vi^	3.7127 (4)

Symmetry codes: (i) 
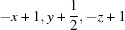
; (ii) 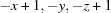; (iii) 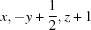; (iv) 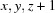; (v) 
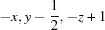
; (vi) 
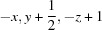
.

**Table 2:** Hydrogen-bond geometry (Å, °)

*D*—H⋯*A*	*D*—H	H⋯*A*	*D*⋯*A*	*D*—H⋯*A*
O1*W*—H11*W*⋯O21^vii^	0.92	2.00	2.905 (3)	168
N4—H42⋯N1^viii^	0.79	2.44	2.985 (5)	127

Symmetry codes: (vii) 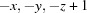; (viii) 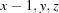.

**Fig. 1.**
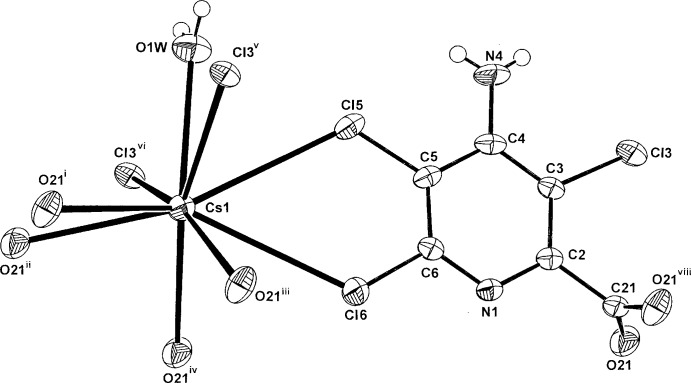
The molecular configuration and atom-numbering scheme for the title compound, with non-H atoms drawn as 50% probability ellipsoids. For symmetry codes: see Table 1.

**Fig. 2.**
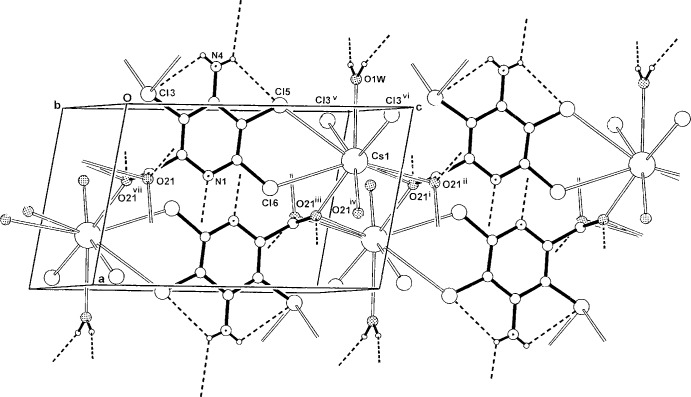
The sheet structure viewed perpendicular to the crystallographic mirror planes, with intermolecular hydrogen bonds and intramolecular N—H···Cl associations shown as dashed lines. For symmetry codes, see Fig. 1 and Table 1.

**Fig. 3.**
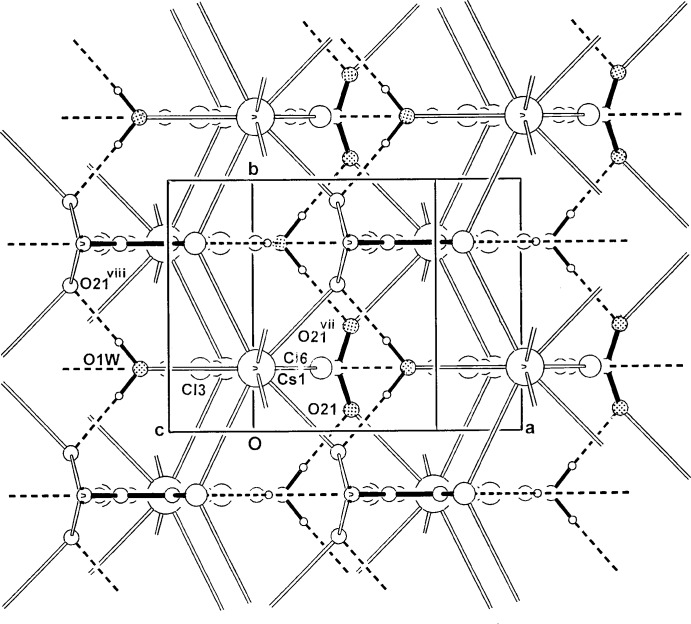
The packing in the unit cell viewed along the the mirror planes showing inter-plane carboxyl bridges and hydrogen-bonding associations (Table 2) as dashed lines.

## supplementary crystallographic information

### Comment

4-Amino-3,5,6-trichloropyridine-2-carboxylic acid (picloram) is a commercial herbicide (Mullinson, 1985) introduced by Dow Chemicals as Tordon (O'Neil, 2001). Although it has potential as a metal chelating ligand similar to picolinic acid, there are only five metal complexes with picloramato ligands in the crystallographic literature. Examples include picloram as a bidentate chelate ligand [with Mn^II^ (Smith *et al.*, 1981*a*) and Cu^II^ (O'Reilly *et al.*, 1983)], while in the Mg complex (Smith *et al.*, 1981*b*], picloram acts as a counter-anion. A caesium complex derived from dipicolinic acid has also been reported (Santra *et al.*, 2011).

The reaction of picloram with caesium hydroxide in aqueous ethanol gave crystals of the title compound [Cs(C_6_H_2_Cl_3_N_2_O_2_)(H_2_O)]_n_ and the structure is reported herein. In this structure, the Cs^+^ cation lies on a crystallographic mirror plane which also contains the coordinating monodentate water molecule and all non-H atoms of the picloramate ligand except the carboxyl O-atom donors (Fig. 1). The irregular CsCl_4_O_5_ coordination sphere comprises chlorine donors from the *ortho*-related ring substituents (Cl5, Cl6) in a bidentate chelate mode [Cs—Cl, 3.6052 (11), 3.7151 (11) Å], with the third chlorine (Cl3) [Cs—Cl, 3.7127 (4) Å] bridging neighbouring Cs^+^ cations [Cs···Cs^x^, Cs···Cs^xi^ = 4.9008 (3) Å] [for (x), -*x* + 1, *y* - 1/2, -*z* + 2; for (xi), -*x* + 1, *y* + 1/2, -*z* + 2], as well as a bidentate chelate and bridging carboxyl group. Although in most structures containing caesium and related ligands, the Cl atom is anionic rather than coordinating, an example of a coordinating carbon-bound Cl is known in which 1,2-dichloroethane acts as a bidentate chelate ligand (Levitskaia *et al.*, 2000). The Cs—Cl bond lengths in that structure are shorter than those in the title complex (3.46–3.56 Å).

In the present complex, sheets are formed parallel to (010) (Fig. 2) and these are extended into a three-dimensional coordination polymer structure through the carboxyl group of the picloram ligand which bridges the sheets down [010] (Fig. 3). The amine group gives weak intramolecular N—H···Cl5 and ···Cl6 interactions and as well forms inter-complex N—H···N_pyridine_ hydrogen bonds which accompany water O—H···O_carboxyl_ hydrogen-bonding interactions in the structure (Table 2).

### Experimental

The title compound was synthesized by heating together under reflux for 10 minutes, 0.5 mmol of caesium hydroxide and 0.5 mmol of 4-amino-3,5,6-trichloropicolinic acid in 20 ml of 10% ethanol–water. Room temperature evaporation of the solution to incipient dryness gave colourless crystal plates of the title complex from which a specimen was cleaved for the X-ray analysis.

### Refinement

Hydrogen atoms of the coordinating water molecule and the amine group were located in a difference-Fourier synthesis but were allowed to ride in the refinement with *U*_iso_(H) = 1.2*U*_eq_(N) or 1.5*U*_eq_(O).

### Crystal data

**Table:**

[Cs(C_6_H_2_Cl_3_N_2_O_2_)(H_2_O)]	*F*(000) = 368
*M**_r_* = 391.37	*D*_x_ = 2.382 Mg m^−^^3^
Monoclinic, *P*2_1_/*m*	Mo *K*α radiation, λ = 0.71073 Å
Hall symbol: -P 2yb	Cell parameters from 3047 reflections
*a* = 7.0816 (3) Å	θ = 3.5–28.7°
*b* = 6.6863 (2) Å	µ = 4.11 mm^−^^1^
*c* = 11.7382 (5) Å	*T* = 200 K
β = 101.005 (4)°	Plate, colourless
*V* = 545.58 (4) Å^3^	0.25 × 0.20 × 0.08 mm
*Z* = 2	

### Data collection

**Table:**

Oxford Diffraction Gemini-S CCD detector diffractometer	1164 independent reflections
Radiation source: Enhance Mo X-ray source	1118 reflections with *I* > 2σ(*I*)
Graphite monochromator	*R*_int_ = 0.026
Detector resolution: 16.077 pixels mm^-1^	θ_max_ = 26.0°, θ_min_ = 3.5°
ω scans	*h* = −8→7
Absorption correction: multi-scan (*CrysAlis PRO*; Agilent, 2012)	*k* = −8→8
*T*_min_ = 0.67, *T*_max_ = 0.98	*l* = −11→14
3773 measured reflections	

### Refinement

**Table:**

Refinement on *F*^2^	Secondary atom site location: difference Fourier map
Least-squares matrix: full	Hydrogen site location: inferred from neighbouring sites
*R*[*F*^2^ > 2σ(*F*^2^)] = 0.021	H-atom parameters constrained
*wR*(*F*^2^) = 0.053	*w* = 1/[σ^2^(*F*_o_^2^) + (0.0338*P*)^2^ + 0.1378*P*] where *P* = (*F*_o_^2^ + 2*F*_c_^2^)/3
*S* = 0.98	(Δ/σ)_max_ = 0.001
1164 reflections	Δρ_max_ = 0.55 e Å^−^^3^
89 parameters	Δρ_min_ = −0.56 e Å^−^^3^
0 restraints	Extinction correction: *SHELXL97* (Sheldrick, 2008) within *WinGX* (Farrugia, 2012), Fc^*^=kFc[1+0.001xFc^2^λ^3^/sin(2θ)]^-1/4^
Primary atom site location: structure-invariant direct methods	Extinction coefficient: 0.0132 (11)

### Special details

**Table:**

Geometry. All e.s.d.'s (except the e.s.d. in the dihedral angle between two l.s. planes) are estimated using the full covariance matrix. The cell e.s.d.'s are taken into account individually in the estimation of e.s.d.'s in distances, angles and torsion angles; correlations between e.s.d.'s in cell parameters are only used when they are defined by crystal symmetry. An approximate (isotropic) treatment of cell e.s.d.'s is used for estimating e.s.d.'s involving l.s. planes.
Refinement. Refinement of *F*^2^ against ALL reflections. The weighted *R*-factor *wR* and goodness of fit *S* are based on *F*^2^, conventional *R*-factors *R* are based on *F*, with *F* set to zero for negative *F*^2^. The threshold expression of *F*^2^ > σ(*F*^2^) is used only for calculating *R*-factors(gt) *etc*. and is not relevant to the choice of reflections for refinement. *R*-factors based on *F*^2^ are statistically about twice as large as those based on *F*, and *R*- factors based on ALL data will be even larger.

### Fractional atomic coordinates and isotropic or equivalent isotropic displacement parameters (Å^2^)

**Table:**

	*x*	*y*	*z*	*U*_iso_*/*U*_eq_	
Cs1	0.29271 (3)	0.2500	0.885412 (19)	0.02831 (13)	
Cl3	−0.06235 (13)	0.2500	0.12344 (9)	0.0326 (2)	
Cl5	−0.01008 (14)	0.2500	0.58974 (9)	0.0291 (2)	
Cl6	0.44747 (14)	0.2500	0.61009 (9)	0.0335 (2)	
O1W	−0.1573 (4)	0.2500	0.8385 (3)	0.0433 (8)	
H11W	−0.2387	0.1425	0.8335	0.065*	
O21	0.4110 (3)	0.0836 (3)	0.14016 (17)	0.0369 (5)	
N1	0.3823 (4)	0.2500	0.3865 (3)	0.0223 (6)	
N4	−0.2153 (5)	0.2500	0.3441 (3)	0.0380 (8)	
H41	−0.2870	0.2500	0.2908	0.046*	
H42	−0.2700	0.2500	0.3968	0.046*	
C2	0.2692 (5)	0.2500	0.2807 (3)	0.0211 (7)	
C3	0.0719 (5)	0.2500	0.2649 (3)	0.0230 (7)	
C4	−0.0239 (5)	0.2500	0.3586 (3)	0.0241 (8)	
C5	0.0961 (5)	0.2500	0.4690 (3)	0.0224 (7)	
C6	0.2937 (5)	0.2500	0.4763 (3)	0.0221 (7)	
C21	0.3725 (5)	0.2500	0.1780 (3)	0.0253 (8)	

### Atomic displacement parameters (Å^2^)

**Table:**

	*U*^11^	*U*^22^	*U*^33^	*U*^12^	*U*^13^	*U*^23^
Cs1	0.02573 (17)	0.03285 (17)	0.02648 (18)	0.000	0.00536 (11)	0.000
Cl3	0.0204 (5)	0.0427 (5)	0.0316 (5)	0.000	−0.0029 (4)	0.000
Cl5	0.0277 (5)	0.0283 (5)	0.0359 (5)	0.000	0.0172 (4)	0.000
Cl6	0.0263 (5)	0.0492 (6)	0.0243 (5)	0.000	0.0034 (4)	0.000
O1W	0.0298 (16)	0.0423 (17)	0.056 (2)	0.000	0.0042 (15)	0.000
O21	0.0456 (12)	0.0340 (11)	0.0352 (12)	0.0137 (9)	0.0178 (10)	0.0035 (9)
N1	0.0173 (15)	0.0238 (15)	0.0266 (17)	0.000	0.0061 (12)	0.000
N4	0.0158 (16)	0.058 (2)	0.041 (2)	0.000	0.0076 (14)	0.000
C2	0.0187 (17)	0.0181 (16)	0.027 (2)	0.000	0.0048 (14)	0.000
C3	0.0169 (17)	0.0240 (17)	0.027 (2)	0.000	0.0008 (15)	0.000
C4	0.0164 (17)	0.0187 (16)	0.037 (2)	0.000	0.0052 (15)	0.000
C5	0.0198 (17)	0.0196 (16)	0.030 (2)	0.000	0.0116 (15)	0.000
C6	0.0194 (17)	0.0216 (16)	0.0243 (19)	0.000	0.0020 (14)	0.000
C21	0.0149 (16)	0.036 (2)	0.0238 (19)	0.000	0.0002 (14)	0.000

### Geometric parameters (Å, º)

**Table:**

Cs1—Cl5	3.7151 (11)	O1W—H11W	0.9200
Cs1—Cl6	3.6052 (11)	O1W—H11W^vii^	0.9200
Cs1—O1W	3.129 (3)	N1—C2	1.343 (5)
Cs1—O21^i^	3.116 (2)	N1—C6	1.326 (5)
Cs1—O21^ii^	3.116 (2)	N4—C4	1.333 (5)
Cs1—O21^iii^	3.150 (2)	N4—H42	0.7900
Cs1—O21^iv^	3.150 (2)	N4—H41	0.7300
Cs1—Cl3^v^	3.7127 (4)	C2—C3	1.374 (5)
Cs1—Cl3^vi^	3.7127 (4)	C2—C21	1.525 (5)
Cl5—C5	1.727 (4)	C3—C4	1.398 (5)
Cl6—C6	1.732 (4)	C3—Cl3	1.749 (4)
C21—O21	1.247 (3)	C4—C5	1.408 (5)
C21—O21^vii^	1.247 (3)	C5—C6	1.386 (5)
			
Cl5—Cs1—Cl6	51.87 (2)	Cl3^vi^—Cs1—O21^iii^	75.19 (4)
Cl5—Cs1—O1W	56.55 (7)	O21^i^—Cs1—O21^ii^	91.43 (5)
Cl5—Cs1—O21^iv^	152.65 (4)	O21^i^—Cs1—O21^iii^	77.10 (5)
Cl3^v^—Cs1—Cl5	78.56 (2)	O21^ii^—Cs1—O21^iii^	106.40 (5)
Cl3^vi^—Cs1—Cl5	78.56 (2)	Cs1^v^—Cl3—C3	100.54 (5)
Cl5—Cs1—O21^i^	100.91 (4)	Cs1^vi^—Cl3—C3	100.54 (5)
Cl5—Cs1—O21^ii^	100.91 (4)	Cs1^v^—Cl3—Cs1^vi^	128.44 (3)
Cl5—Cs1—O21^iii^	152.65 (4)	Cs1—Cl5—C5	120.18 (13)
Cl6—Cs1—O1W	108.42 (7)	Cs1—Cl6—C6	124.53 (13)
Cl6—Cs1—O21^iv^	141.22 (4)	Cs1^viii^—O21—Cs1^ix^	102.90 (6)
Cl3^v^—Cs1—Cl6	100.49 (2)	Cs1—O1W—H11W	128.00
Cl3^vi^—Cs1—Cl6	100.49 (2)	Cs1—O1W—H11W^vii^	128.00
Cl6—Cs1—O21^i^	65.68 (4)	H11W—O1W—H11W^vii^	103.00
Cl6—Cs1—O21^ii^	65.68 (4)	C2—N1—C6	116.5 (3)
Cl6—Cs1—O21^iii^	141.22 (4)	C4—N4—H41	130.00
O1W—Cs1—O21^iv^	104.15 (7)	H41—N4—H42	108.00
Cl3^v^—Cs1—O1W	64.39 (1)	C4—N4—H42	123.00
Cl3^vi^—Cs1—O1W	64.39 (1)	N1—C2—C21	116.1 (3)
O1W—Cs1—O21^i^	131.69 (4)	N1—C2—C3	122.4 (3)
O1W—Cs1—O21^ii^	131.69 (4)	C3—C2—C21	121.5 (3)
O1W—Cs1—O21^iii^	104.15 (7)	C2—C3—C4	121.8 (3)
Cl3^v^—Cs1—O21^iv^	75.19 (4)	Cl3—C3—C2	118.9 (3)
Cl3^vi^—Cs1—O21^iv^	112.36 (4)	Cl3—C3—C4	119.3 (3)
O21^iv^—Cs1—O21^i^	106.40 (5)	N4—C4—C5	122.5 (3)
O21^iv^—Cs1—O21^ii^	77.10 (5)	C3—C4—C5	115.2 (3)
O21^iv^—Cs1—O21^iii^	41.36 (5)	N4—C4—C3	122.3 (3)
Cl3^v^—Cs1—Cl3^vi^	128.44 (2)	C4—C5—C6	118.8 (3)
Cl3^v^—Cs1—O21^i^	160.44 (4)	Cl5—C5—C4	118.4 (3)
Cl3^v^—Cs1—O21^ii^	69.70 (4)	Cl5—C5—C6	122.8 (3)
Cl3^v^—Cs1—O21^iii^	112.36 (4)	Cl6—C6—C5	120.6 (3)
Cl3^vi^—Cs1—O21^i^	69.70 (4)	Cl6—C6—N1	114.2 (3)
Cl3^vi^—Cs1—O21^ii^	160.44 (4)	N1—C6—C5	125.2 (3)
			
Cl6—Cs1—Cl5—C5	0.00 (1)	C21—C2—C3—C4	180.00 (1)
O1W—Cs1—Cl5—C5	180.00 (1)	N1—C2—C21—O21	89.9 (3)
Cl5—Cs1—Cl6—C6	0.00 (1)	C3—C2—C21—O21	−90.1 (3)
O1W—Cs1—Cl6—C6	0.00 (1)	Cl3—C3—C4—N4	0.00 (1)
Cs1—Cl5—C5—C4	180.00 (1)	Cl3—C3—C4—C5	180.00 (1)
Cs1—Cl5—C5—C6	0.00 (1)	C2—C3—C4—N4	180.00 (1)
Cs1—Cl6—C6—N1	180.00 (1)	C2—C3—C4—C5	0.00 (1)
Cs1—Cl6—C6—C5	0.00 (1)	N4—C4—C5—Cl5	0.00 (1)
C6—N1—C2—C3	0.00 (1)	N4—C4—C5—C6	180.00 (1)
C6—N1—C2—C21	180.00 (1)	C3—C4—C5—Cl5	180.00 (1)
C2—N1—C6—Cl6	180.00 (1)	C3—C4—C5—C6	0.00 (1)
C2—N1—C6—C5	0.00 (1)	Cl5—C5—C6—Cl6	0.00 (1)
N1—C2—C3—Cl3	180.00 (1)	Cl5—C5—C6—N1	180.00 (1)
N1—C2—C3—C4	0.00 (1)	C4—C5—C6—Cl6	180.00 (1)
C21—C2—C3—Cl3	0.00 (1)	C4—C5—C6—N1	0.00 (1)

Symmetry codes: (i) −*x*+1, *y*+1/2, −*z*+1; (ii) −*x*+1, −*y*, −*z*+1; (iii) *x*, −*y*+1/2, *z*+1; (iv) *x*, *y*, *z*+1; (v) −*x*, *y*−1/2, −*z*+1; (vi) −*x*, *y*+1/2, −*z*+1; (vii) *x*, −*y*+1/2, *z*; (viii) *x*, *y*, *z*−1; (ix) −*x*+1, *y*−1/2, −*z*+1.

### Hydrogen-bond geometry (Å, º)

**Table:**

*D*—H···*A*	*D*—H	H···*A*	*D*···*A*	*D*—H···*A*
O1*W*—H11*W*···O21^x^	0.92	2.00	2.905 (3)	168
N4—H42···N1^xi^	0.79	2.44	2.985 (5)	127
N4—H42···Cl5	0.79	2.63	2.971 (4)	108
N4—H41···Cl3	0.73	2.75	2.992 (4)	102

Symmetry codes: (x) −*x*, −*y*, −*z*+1; (xi) *x*−1, *y*, *z*.
